# Delayed atorvastatin delivery promotes recovery after experimental spinal cord injury

**DOI:** 10.1016/j.neurot.2024.e00517

**Published:** 2025-01-04

**Authors:** Samuel C. Buchl, Ha Neui Kim, Benjamin Hur, Whitney L. Simon, Monica R. Langley, Jaeyun Sung, Isobel A. Scarisbrick

**Affiliations:** a Mayo Clinic Graduate School of Biomedical Sciences, Rochester, MN 55905, USA; b Department of Physical Medicine and Rehabilitation, Mayo Clinic, Rochester, MN, USA; c Microbiomics Program, Center for Individualized Medicine, Mayo Clinic, Rochester, MN, USA; d Division of Computational Biology, Department of Quantitative Health Sciences, Mayo Clinic, Rochester, MN, USA; e Center for Multiple Sclerosis and Autoimmune Neurology, Mayo Clinic, Rochester, MN, USA; f Division of Rheumatology, Department of Medicine, Mayo Clinic, Rochester, MN, USA

**Keywords:** Chronic spinal cord injury, Locomotor recovery, RNA sequencing, Spinal cord transcriptomics, Pathway analysis

## Abstract

Spinal cord injury (SCI) significantly alters gene expression, potentially impeding functional recovery. This study investigated the effects of atorvastatin, a widely prescribed cholesterol-lowering drug, on gene expression and functional recovery in a chronic murine SCI model. Female C57BL/6J mice underwent moderate 0.25 ​mm lateral compression SCI and received daily atorvastatin (10 ​mg/kg) or vehicle-only injections from two weeks post-injury for four weeks. Sensorimotor functions were assessed using the Basso Mouse Scale (BMS), its subscore, and the inclined plane test. RNA sequencing of spinal cord tissues identified robust transcriptomic changes from SCI and a smaller subset from atorvastatin treatment. Atorvastatin enhanced sensorimotor recovery within two weeks of treatment initiation, with effects persisting to the experimental endpoint. Pathway analysis showed atorvastatin enriched neural regeneration processes including Fatty Acid Transport, Axon Guidance, and the Endocannabinoid Developing Neuron Pathway; improved mitochondrial function via increased TCA Cycle II and reduced Mitochondrial Dysfunction; and decreased Inhibition of Matrix Metalloproteases. Key gene drivers included *Fabp7*, *Unc5c*, *Rest*, and *Klf4*. Together, these results indicate atorvastatin's potential in chronic SCI recovery, especially where already indicated for cardiovascular protection.

## Graphical abstract

**Figure:**
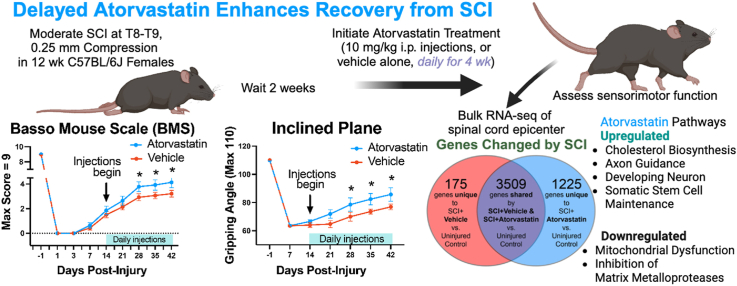

## Introduction

In the landscape of cardiovascular therapeutics, statins (3-Hydroxy-3-methylglutaryl coenzyme A reductase [HMGCR] inhibitors), are widely prescribed for their critical role in lipid disorder management [1]. Atorvastatin is one statin particularly notable for its ability to reduce blood cholesterol levels and cardiovascular disease risk [2,3] and is overall one of the most prescribed pharmaceuticals in the United States year-to-year [4]. Amid rising cardiovascular disease rates, with 40% of the U.S. population estimated to be impacted by 2030 [5,6], atorvastatin's high prescription rate reflects its critical therapeutic relevance. Atorvastatin use is particularly important for individuals with severe spinal cord injuries (SCI), whose reduced mobility further elevates cardiovascular risk [[7], [8], [9]]. Despite atorvastatin’s benefits, including reduced overall mortality rates for those with SCI [10], its usage raises concerns, particularly regarding central nervous system (CNS) health.

As a highly lipophilic statin, atorvastatin inherently crosses the intact blood-brain barrier (BBB) [11], potentially leading to off-target impacts. These concerns intensify in cases of CNS disease and injury, where a compromised BBB allows greater drug penetration [[12], [13], [14], [15]]. This increased penetrance may explain some reports of cognitive dysfunction that led to the FDA's black box warning for the drug [16]. Since the CNS depends on endogenous cholesterol synthesis [[17], [18], [19]], especially following injury [20], understanding the role of atorvastatin, particularly within the injured CNS, is of high clinical relevance.

Recent studies offer contrasting insights into the effects of statin treatment when initiated before or acutely following CNS injuries. While some studies indicate enhanced recovery [[21], [22], [23], [24], [25], [26], [27], [28], [29], [30], [31], [32]], others report limited or no significant benefit [[33], [34], [35]], or even impaired remyelination [36]. Our own retrospective analysis of a historical cohort of individuals with SCI indicated that use of statins, including atorvastatin, initiated before SCI and continuing through the acute recovery phase correlated with reduced improvements in motor function over the first two months following SCI [37]. An earlier study showed that individuals with hyperlipidemia and lumbar SCI experienced modest protection from low-dose but not high-dose atorvastatin treatment [38]. Together these findings stress the urgency for a mechanistic understanding of statins’ actions in the injured CNS, and the conditions under which statins hinder or enhance recovery.

In the present study, we determined the impact of atorvastatin delivery initiated 2 ​wk after experimental murine SCI on sensorimotor recovery and on gene expression patterns in the injured spinal cord. Our findings uncover a beneficial role for atorvastatin after SCI, demonstrating enhancements in sensorimotor recovery and modulation of SCI-induced transcriptional changes.

## Materials and Methods

### Overview of SCI lateral compression model and post-SCI treatments

Twelve-week-old female C57BL/6J mice (stock #000664, Jackson Laboratory, Bar Harbor, ME) were utilized for all SCI procedures. Mice were maintained in an environmentally controlled setting, with temperatures regulated between 22 and 24C and a consistent 12-h light/dark cycle. All experimental protocols were conducted following approval from the Mayo Clinic Institutional Animal Care and Use Committee (IACUC) and in compliance with National Institutes of Health guidelines.

**Fig. 1:**
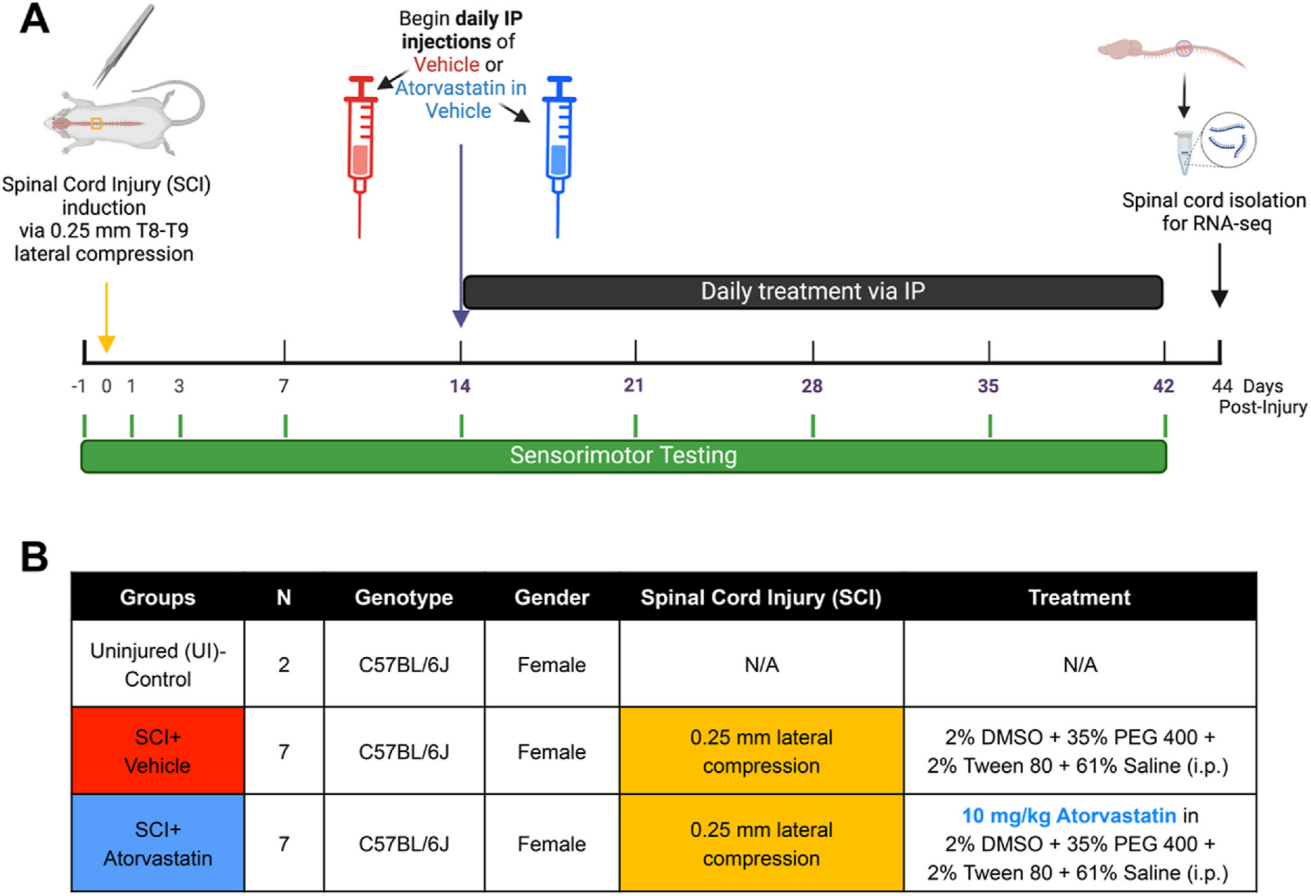
**Experimental design for delayed atorvastatin or vehicle control administration following lateral compression SCI.** (A) Experimental timeline for evaluating the effects of atorvastatin on SCI in C57BL/6J female mice. (B) Table of experimental groups (sex, n included in table) for assessing the therapeutic effect of atorvastatin delivery initiated 2 ​wk post-SCI. Experimental mice underwent a laminectomy at vertebral T8-T9 followed by lateral compression SCI using forceps with a 0.25 ​mm gap. Subsequently mice were randomized across two groups: SCI ​+ ​Atorvastatin or SCI ​+ ​Vehicle, receiving, respectively, daily intraperitoneal (i.p.) injections of either atorvastatin in a vehicle, or the vehicle alone, starting 14 ​d post-injury. Daily injections continued for 4 ​wk, with sensorimotor recovery evaluated using BMS and inclined plane tests at specified intervals (1A, green ticks). SCI was induced in the experimental groups when mice were 12 ​wk. Spinal cords from Uninjured Control mice were isolated at 14 ​wk of age. In the SCI groups, treatment (atorvastatin or vehicle) commenced when mice were 14 ​wk old and continued until spinal cord isolation at 18 ​wk of age for downstream RNA-seq analyses.

The experimental timeline is shown in Fig. 1A. During the initial phase of the study, age-matched female mice were randomized into two SCI conditions. The first condition, designated the SCI ​+ ​Atorvastatin group, included 7 mice subjected to lateral compression SCI and subsequent intraperitoneal (i.p.) delivery of atorvastatin (10 ​mg/kg, Sigma-Aldrich) dissolved in a vehicle composed of 2 ​% dimethyl sulfoxide (DMSO; ATCC), 35 ​% polyethylene glycol (PEG) 400 (Sigma-Aldrich), 2 ​% Tween 80 (Sigma-Aldrich), and 61 ​% saline. The second condition, designated the SCI ​+ ​Vehicle group, also included 7 mice subjected to SCI, followed by i.p. injections of the vehicle alone.

Sample size determination was informed by variability in Basso Mouse Scale (BMS) scores from prior SCI research [39,40]. A power analysis, conducted for ANOVA with a target statistical power of 0.8 and a significance level of α ​= ​0.05 using SigmaStat software version 13.0 (Systat Software Inc.), indicated that at least six mice per group would be necessary to reliably detect a 2-point difference in mean BMS scores, a functionally significant difference in terms of motor function. To account for potential losses due to mortality, one mouse was added to each group, for n ​= ​7 per SCI group. Additionally, two female C57BL/6J mice, aged 14 ​wk at the time of spinal cord isolation served as Uninjured Controls for subsequent RNA-seq analysis. Fig. 1B displays a table outlining all mice and conditions included in this study.

In this study, we employed a moderate lateral compression SCI model, known to induce substantial astrocytic and microglial activation as well as neuronal death, which contribute to incomplete recovery [[41], [42], [43], [44], [45], [46], [47]]. Deep anesthesia was induced in the mice via i.p. injection of xylazine (10 ​mg/kg, Akorn, Inc., Lake Forest, IL) and ketamine (100 ​mg/kg, Fort Dodge, IA). To mitigate the risk of infection, Baytril (10 ​mg/kg, Bayer Health Care, Shawnee Mission, KS) was administered i.p. prior to the surgical procedure. A precise laminectomy was performed at the T8-9 vertebral level, ensuring the dura mater remained intact. Subsequently, Dumont-type 2 forceps equipped with a 0.25 ​mm spacer were employed to laterally compress the spinal cord for a duration of 14 ​s [46,47]. Post-surgery, each mouse received 0.2 ​ml saline i.p. to replenish blood volume. Recovery was facilitated on a heating pad, accompanied by daily i.p. Buprenex injections (0.05 ​mg/kg, Reckitt Benckiser Healthcare, England) every 12 ​h for 72 ​h post-surgery, following an initial preoperative dose, to alleviate pain. Manual bladder voiding was performed twice daily until the restoration of autonomous bladder function. Throughout the experiment, mice had unrestricted access to food and water.

### Randomization and treatment schedule

At 14 ​d post-injury, the 14-week-old mice with SCI underwent sensorimotor testing and body mass measurement and were randomized into treatment groups: SCI ​+ ​Atorvastatin or SCI ​+ ​Vehicle. Treatment groups were subsequently confirmed to have statistically equivalent scores on all sensorimotor tests and equivalent body mass at this time point (unpaired two-tailed t-tests, all ps ​> ​0.05). Daily i.p injections were then initiated by a blinded experimenter without knowledge of the specific treatment being administered. Treatment continued for 4 ​wk (Fig. 1A-B).

Atorvastatin and vehicle were administered i.p. to ensure consistent delivery, minimize stress and potential injury from repeated oral gavage [48], and to maintain consistency with prior studies [25,26,29,32]. This route mitigates issues with gastrointestinal absorption due to SCI-induced dysfunction [49] and reduces variability associated with oral administration [50]. The dose of 10 ​mg/kg was chosen based on prior dose-response studies in rodent neuropathic pain models, which identified maximal efficacy at this dose compared to lower (3 ​mg/kg) or higher (30 ​mg/kg) doses [51,52].

### Sensorimotor outcome measures

**Fig. 2:**
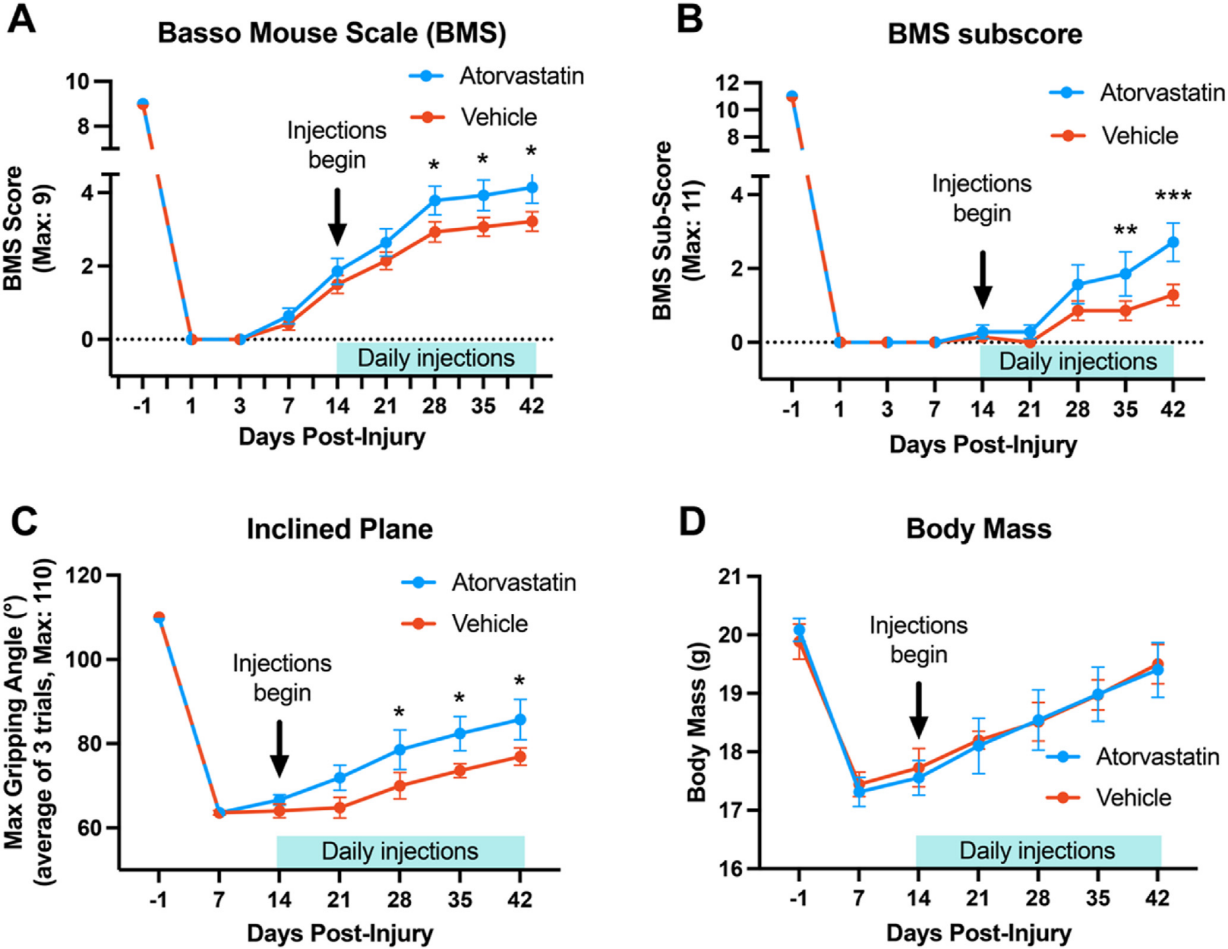
**Delayed atorvastatin treatment enhances functional recovery following SCI.** Measures of sensorimotor function and body mass were conducted pre-injury and at multiple post-injury time points. (A) Basso Mouse Scale (BMS) scores highlight improved locomotor recovery in atorvastatin-treated mice, indicating significant enhancements in movements such as ankle motion and tail position starting from 2 ​wk post-treatment, with elevated scores at 28, 35, and 42 ​d post-injury. (B) BMS subscores indicate enhanced recovery of additional motor measures, including stepping frequency, coordination, and trunk stability, at 35 and 42 ​d post-injury. (C) Inclined plane test results demonstrate increased strength in atorvastatin-treated mice, with notable improvements in ability to maintain a grip at greater angles observed at 28, 35, and 42 ​d post-injury. (D) Body mass measurement, an indirect measure of health and recovery, showed no significant differences between groups throughout the recovery period. Statistical analysis utilized two-way repeated measures ANOVA and Student-Newman-Keuls post-hoc tests. Values represent mean ​± ​SEM. ∗∗∗p ​< ​0.001; ∗∗p ​< ​0.01; ∗p ​< ​0.05.

To assess sensorimotor function, the Basso Mouse Scale (BMS) open field test was utilized prior to injury (d −1), at 1, 3, and 7 ​d after SCI, and weekly thereafter to 42 ​d after injury. As previously described [40,46], mice were placed in a circular, Plexiglas-enclosed open field in which movements were video-recorded for a period of 3 ​min. Subsequently, a blinded observer analyzed various aspects of motor function, including paw placement, parallel paw position and forelimb-hindlimb coordination. This analysis yielded the BMS score with a maximum of 9 (Fig. 2A). Additionally, the BMS subscore was used to assess changes in stepping frequency, coordination, paw position, trunk stability, and tail position, yielding a maximum score of 11 (Fig. 2B). The inclined plane test, serving as a second independent assessment of sensorimotor function, was administered pre-injury (d −1), 7 days post-SCI, and then weekly until 42 days post-injury (Fig. 2C). This test evaluates sensorimotor control and grip strength by measuring the maximum angle at which a mouse can maintain its grip on a textured surface, with the incline adjusted gradually up to a maximum of 110° from the horizontal position [53,54]. Body mass was also measured on d −1, d 7, then weekly to 42 ​d post-injury to assess feeding behavior and overall health (Fig. 2D).

### Isolation of RNA from tissues

**Fig. 3:**
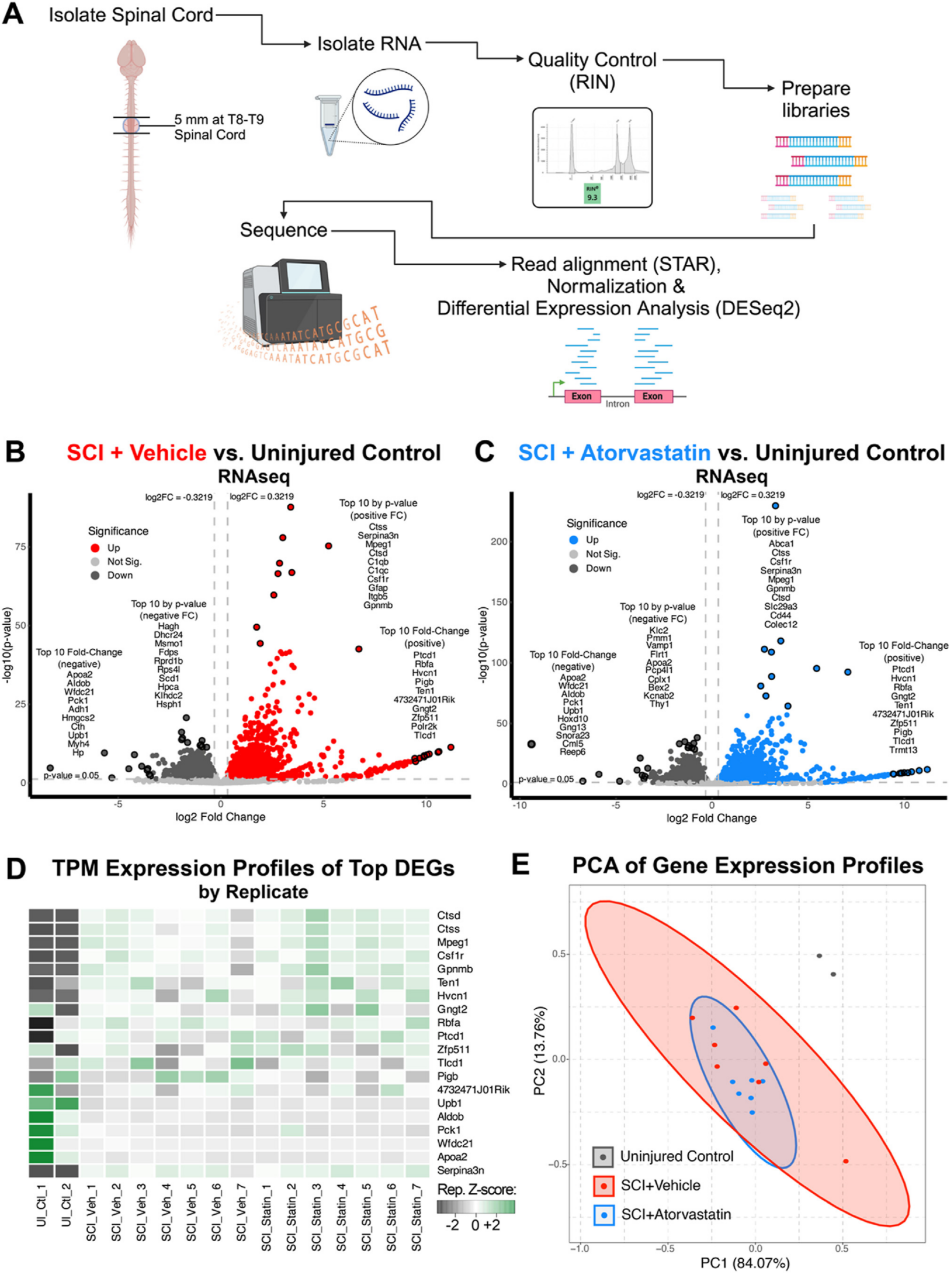
**Gene expression profiling of spinal cord shows a common transcriptomic response across SCI treatment conditions and an effect of delayed delivery of atorvastatin.** (A) Spinal cord sample processing workflow depicting spinal cord isolation to RNA sequencing at 44 ​d post-injury. A 5 ​mm segment at the injury epicenter (vertebral levels T8-T9) was collected from the SCI-treated mice and the corresponding region from Uninjured Controls. The samples underwent RNA extraction, quality assessment, library construction, and high-throughput Illumina paired-end sequencing. “RIN” ​= ​RNA integrity number. (B) Volcano plots compare gene expression of SCI ​+ ​Vehicle with Uninjured Controls or (C) SCI ​+ ​Atorvastatin with Uninjured Controls, highlighting differentially expressed genes surpassing the expression fold change and statistical significance thresholds (|log2(fold change)| ​> ​0.322 [>25 ​% change]; FDR <0.05), with top 10 genes by fold change and p value in each quadrant encircled and listed. (D) Heatmap of gene expression by replicate in transcripts per million (TPM) details genes commonly changed in SCI across both treatment groups, indicating a core set of SCI-responsive genes. The Z-score for each replicate was calculated across groups per gene. “UI_Ctl” ​= ​Uninjured Control; “SCI_Veh” ​= ​SCI ​+ ​Vehicle; “SCI_Statin” ​= ​SCI ​+ ​Atorvastatin. (E) PCA plot delineates the groups based on gene expression, with the SCI ​+ ​Atorvastatin group showing tighter clustering.

At the experimental endpoint 44 ​d post-injury, mice were injected with a terminal dose of pentobarbital (60 ​mg/kg; Abbott Laboratories), perfused with ice-cold saline, and spinal cords were isolated. To provide an additional control for RNA-sequencing (RNA-seq), spinal cords from the sex-matched Uninjured Control mice (n ​= ​2) were harvested in parallel. The complete tissue isolation and RNA-seq pipeline is depicted in Fig. 3A.

Immediately following dissection, a 5 ​mm section of the spinal cord centered around the injury site of injured mice or the equivalent region in uninjured mice was preserved in RNAlater (Invitrogen) according to the manufacturer's protocol and then stored at −80 ​°C. RNA extraction was carried out using Trizol (Invitrogen), followed by a purification step with the RNeasy Mini Kit (Qiagen), which included an on-column DNase treatment to eliminate genomic DNA. Quantity and quality of the extracted RNA were assessed using RiboGreen (Thermo Fisher Scientific) for quantification and the High Sensitivity RNA ScreenTape assay (TapeStation 4.1.1, Agilent) for quality control. Library preparation was conducted using the SMARTer Stranded Total RNA-seq Kit v2 - Pico Input Mammalian (Takara Bio), with subsequent normalization and pooling for sequencing. Sequencing was conducted at the University of Minnesota Genomics Center (Minneapolis, MN), on the Illumina NovaSeq 6000 System using a flow cell for 150 base-pair paired-end reads, targeting a depth of 40 million reads per sample.

To ensure robust detection of differential gene expression resulting from atorvastatin treatment within context of SCI, we allocated the majority of sequencing reads to the SCI groups (n ​= ​7 each for SCI ​+ ​Atorvastatin and SCI ​+ ​Vehicle control), as informed by group sizes identified by the power analysis to detect significant sensorimotor differences. The two remaining slots in the S4 Flow Cells on the Illumina NovaSeq 6000 were allocated to RNA from the same spinal cord region of the 2 Uninjured Control mice.

### RNA-seq data pre-processing and read alignment

The RNA-seq processing pipeline began with the assessment of sequence quality for the generated paired-end reads (.fastq files) using FASTQC (version 0.12.1), where all files passed without quality concerns [55]. Paired-end raw reads were trimmed by Trimmomatic (version 0.38) with the following parameter: ILLUMINACLIP:TruSeq3-PE:2:30:10. Subsequent alignment of the trimmed reads to the mouse reference genome (mm10) was accomplished using STAR (version 2.5.4b). For quantification of gene expression, RSEM (version 1.3.1; with the parameter –star-sjdboverhang 150) processed the .bam files produced by STAR to calculate transcripts per million (TPM). Gene annotations for Mouse Genome Assembly version 10 (mm10) were sourced from the UCSC Genome Browser (2023, genome.ucsc.edu).

### Investigation of global transcriptome variance

Transcriptomes were composed of the log_2_-transformed TPM (with a pseudocount addition of 0.001) values of 24,411 genes. Among 16 samples (SCI ​+ ​Atorvastatin, n ​= ​7; SCI ​+ ​Vehicle, n ​= ​7; Uninjured Control, n ​= ​2), genes with an average per-condition TPM value above 1 (n ​= ​16,092) [56,57] were projected onto a principal component analysis (PCA) ordination plot.

### Identification of DEGs

DESeq2 (version 1.26.0) [55] was used to identify differentially expressed genes (DEGs) between groups: SCI ​+ ​Atorvastatin (n ​= ​7), SCI ​+ ​Vehicle (n ​= ​7), and Uninjured Control (n ​= ​2). Only genes with a condition-mean TPM greater than 1 were included for further analyses [56,57]. In this study, we utilized a two-tiered approach to identify DEGs in the context of SCI, and to elucidate the specific genomic effects of atorvastatin treatment.

Initially, our analysis aimed to distinguish DEGs in SCI conditions compared to an Uninjured Control group. The criterion for differential expression was established as |log_2_(fold change)| ​> ​0.322 indicative of gene expression alterations exceeding 25%. To minimize the risk of type I error in multiple comparisons, p values were adjusted using the Benjamini-Hochberg procedure [58] to control the False Discovery Rate (FDR) at α ​= ​0.05.

Subsequently, we focused on discerning the specific transcriptional impact of atorvastatin in the SCI context. This involved directly comparing gene expression profiles of SCI ​+ ​Atorvastatin mice and SCI ​+ ​Vehicle mice. The same |log_2_(fold change)| ​> ​0.322 threshold was used to identify expression changes of a biologically significant magnitude. For this comparison, we implemented a relaxed significance threshold of p ​< ​0.001, given that the DEGs of interest had already satisfied a stringent control for multiple testing corrections (FDR <0.05) in the initial analysis against the Uninjured Control group.

### Functional annotation and gene set enrichment analysis

We next conducted an in-depth functional annotation and gene set enrichment analysis to further understand the biological implications of the identified DEGs. Both upregulated and downregulated DEGs were analyzed for pathway associations using three bioinformatics platforms: Ingenuity Pathway Analysis (IPA, version 01-22-01, Qiagen), the Protein Analysis Through Evolutionary Relationships (PANTHER, version 16.0, Global Core Biodata Resource) database, and the Database for Annotation, Visualization and Integrated Discovery (DAVID, v2024q2, https://david.ncifcrf.gov/). Associations between DEGs and pathways were deemed statistically significant at p ​< ​0.05 across all platforms.

### Statistical analysis for randomization and treatment effects

Sensorimotor data, including Basso Mouse Scale (BMS) scores, BMS subscores, inclined plane test scores, and body mass measurements, are presented as mean ​± ​standard error of the mean (SEM). Initially, unpaired two-tailed t-tests were utilized to ensure balanced randomization of treatment groups with no significant differences in sensorimotor scores or body mass at 14 ​d post-injury. Subsequently, Two-Way Analysis of Variance (ANOVA), performed with SigmaStat software version 13.0 (Systat Software) assessed the influence of atorvastatin on sensorimotor recovery over time. This analysis included fixed effects for treatment and time, and was applied to BMS scores, BMS subscores, and inclined plane test scores. Post-hoc comparisons were conducted using the Student-Newman-Keuls (SNK) test, with a significance threshold set at p ​< ​0.05 across all tests.

## Results

### Delayed atorvastatin delivery promotes sensorimotor recovery following SCI

Mice receiving daily atorvastatin for 4 ​wk starting 2 ​wk after SCI showed improved locomotor recovery in the BMS open field test, as evidenced by the total score (Fig. 2A, Two-Way Repeated Measures ANOVA, SNK, p ​< ​0.001, day *F*_(8,108)_ ​= ​237.180, treatment *F*_(1,108)_ ​= ​11.706, SCI ​+ ​Atorvastatin *n* ​= ​7, SCI ​+ ​Vehicle *n* ​= ​7). When examining the BMS subscore, which evaluates stepping frequency, coordination, and paw position, atorvastatin-treated mice displayed significantly better recovery at the final two measurement time points (SNK post-hoc analyses: 35 ​d post-injury, p ​< ​0.008; 42 ​d post-injury, p ​< ​0.001).

Mice treated with atorvastatin also demonstrated enhanced performance in maintaining their grip on the inclined plane test (Fig. 2C, Two-Way Repeated Measures ANOVA: F_(1,84)_ for treatment ​= ​13.195, p ​< ​0.001), indicating increased strength and/or motor control. Post hoc comparisons with SNK showed significant differences at 28 (p ​< ​0.03), 35 (p ​< ​0.03), and 42 ​d after SCI (p ​< ​0.03). No differences were observed in body mass across time points (Fig. 2D).

### SCI modulates a core gene set across injury conditions

Following the isolation and sequencing of spinal cord RNA along with subsequent identification of DEGs (Fig. 3A), we conducted two key comparisons to investigate SCI and SCI ​+ ​Atorvastatin associated gene expression changes. First, we contrasted spinal cords from SCI mice receiving the vehicle treatment (SCI ​+ ​Vehicle) or atorvastatin (SCI ​+ ​Atorvastatin) against those from Uninjured Control mice. For each comparison (SCI condition vs. Uninjured Control), differential gene expression analysis was performed and DEGs visualized using volcano plots with log_2_(fold change) expression plotted against corresponding p values (Fig. 3B-C). For an initial comparison of DEGs across SCI conditions, we enumerated the 10 most upregulated and 10 most downregulated DEGs from each comparison (SCI condition vs. Uninjured) based on fold change, as well as the 10 upregulated and 10 downregulated DEGs with the most significant p values in each category. This approach yielded a set of 40 genes for each comparison: SCI ​+ ​Vehicle vs. Uninjured Control (Fig. 3B) and SCI ​+ ​Atorvastatin vs. Uninjured Control (Fig. 3C). All genes and TPM expression values are shown in Supplementary Table 1. A subsequent cross-referencing of these gene sets revealed a subset of 20 genes (15 upregulated, 5 downregulated) that were consistently identified as significantly changed in both SCI conditions (Fig. 3D). The overlap of half (20 of 40) of the top DEGs from each comparison indicates a core transcriptional response at 44 ​d after SCI evident irrespective of treatment condition.

Principal component analysis (PCA) was subsequently applied to project the transcriptomic profiles onto an ordination plot (Fig. 3E). PCA revealed distinct clustering patterns, with both the SCI ​+ ​Atorvastatin and SCI ​+ ​Vehicle groups forming separate clusters from the Uninjured Control group, while the SCI groups exhibited significant overlap regardless of treatment. Within the SCI ​+ ​Atorvastatin group, however, there was slightly tighter clustering compared to the SCI ​+ ​Vehicle group, suggesting a more uniform transcriptional response. This finding indicates a potential stabilizing effect of atorvastatin on gene expression patterns when delivered at a chronic time point after SCI.

### Atorvastatin modulates a gene set responsive to SCI

**Fig. 4:**
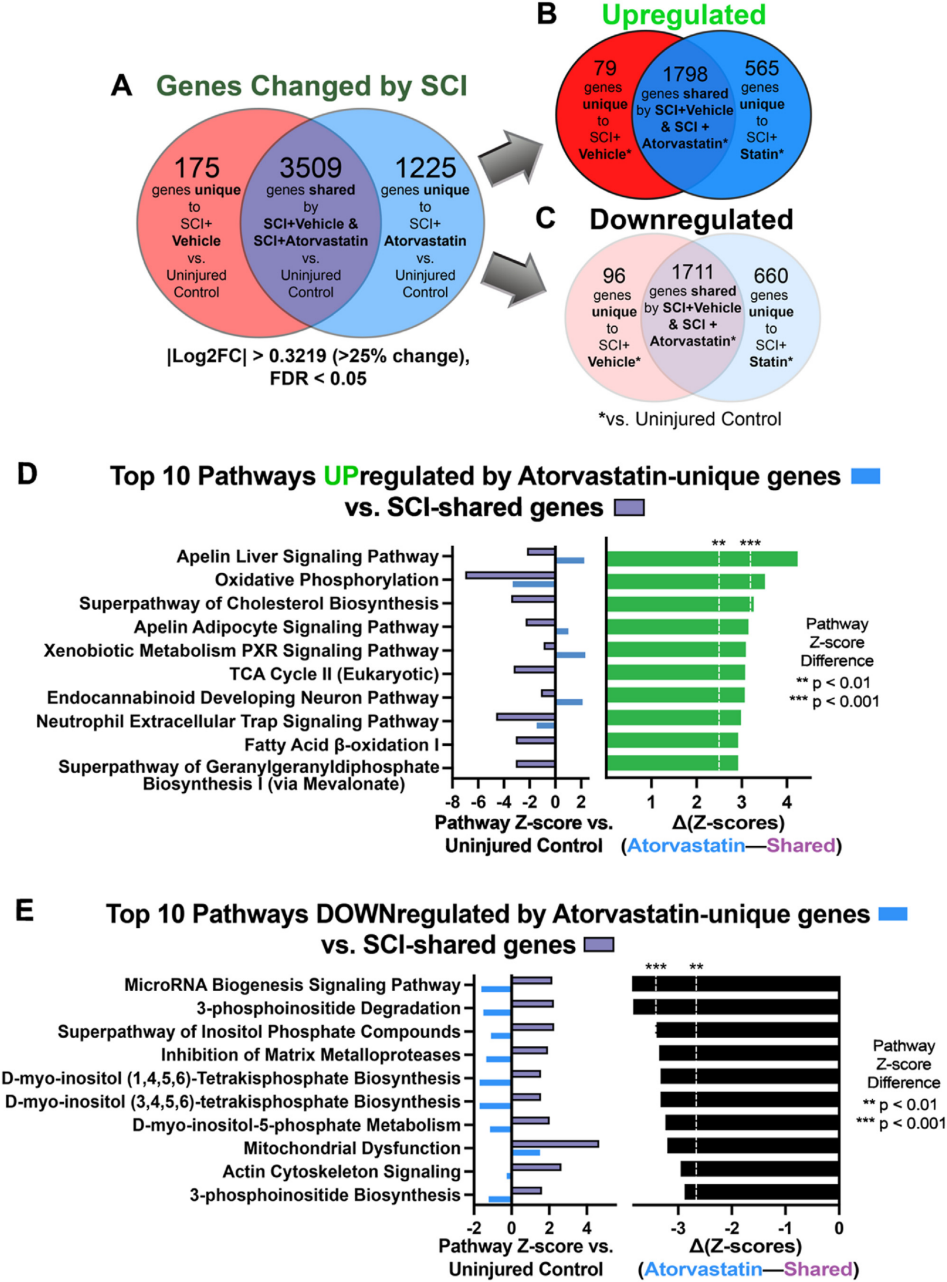
**Delayed atorvastatin delivery modulates differential gene expression and pathway engagement after SCI.** (A) Venn diagram depicting overall expression changes following SCI, with comparisons of genes commonly and uniquely affected by atorvastatin or vehicle treatments relative to the Uninjured Control. (B) Genes upregulated or (C) downregulated by SCI, illustrating the unique and shared transcriptional changes elicited by atorvastatin vs. vehicle treatment when compared to uninjured control. Abbreviation in (B) and (C): “SCI ​+ ​Statin” ​= ​SCI ​+ ​Atorvastatin. These analyses quantify the scale of transcriptional modifications induced by SCI and the extent to which atorvastatin treatment influences gene expression, utilizing a |log_2_(fold change)| threshold >0.322 (>25% absolute change) and FDR <0.05. (D and E) Ingenuity Pathway Analysis (IPA) of pathways significantly affected by atorvastatin treatment, comparing “SCI-shared” pathways (common differentially expressed genes (DEGs) from both SCI ​+ ​Atorvastatin and SCI ​+ ​Vehicle conditions vs. Uninjured Control) to "Atorvastatin-unique" pathways (DEGs specific to SCI ​+ ​Atorvastatin vs. Uninjured Control). Pathways listed showed significantly different activation between SCI-shared and Atorvastatin-unique gene sets, ranked by Z-score difference. (D) Top 10 upregulated pathways sorted by descending Z-score difference. (E) Top 10 downregulated pathways sorted by ascending Z-score difference. ∗∗∗Δ|Z-score| > 3.29: p ​< ​0.001; ∗∗Δ|Z-score| > 2.58: p ​< ​0.01.

We next compared the specific gene expression changes triggered by SCI to those elicited by atorvastatin and vehicle treatments (Fig. 4A–C). Comparisons involved three key groups: (1) genes altered by both SCI conditions; (2) genes uniquely changed by SCI ​+ ​Vehicle; and (3) genes uniquely changed by SCI ​+ ​Atorvastatin, all in comparison to the Uninjured Control group (|log_2_(fold change)| ​> ​0.322 indicating a statistically significant change in expression ≥25 ​%, FDR <0.05). We identified a seven-fold increase in the number of genes uniquely modulated by the SCI ​+ ​Atorvastatin treatment (1225 genes) compared to those altered in the SCI ​+ ​Vehicle group (175 genes) (p ​< ​0.001, binomial test; Fig. 4A), demonstrating atorvastatin's significant modulatory effect on the chronically injured spinal cord compared to the vehicle control.

To elucidate the impact of SCI and delayed atorvastatin treatment on gene expression, we divided the DEGs into two distinct categories: upregulated and downregulated genes in SCI conditions. This categorization (Fig. 4B: upregulated DEGs; Fig. 4C: downregulated DEGs), was undertaken to determine whether SCI or atorvastatin treatment predominantly activates or inhibits gene expression and elucidate possible treatment effects on the transcriptome. Our analysis revealed overall decreased expression with delayed delivery of atorvastatin after SCI, with 660 genes downregulated compared to 565 genes upregulated (p ​< ​0.008, binomial test). Conversely, the delivery of vehicle did not demonstrate a significant difference between upregulated and downregulated genes (96 vs. 79, respectively; p ​= ​0.226, binomial test). Notably, when examining the subset of genes differentially expressed at 44 ​d post-SCI in both atorvastatin and vehicle treated mice, we observed a near-equal distribution of upregulated and downregulated genes (1798 vs. 1711, respectively; p ​= ​0.147, binomial test).

### Delayed atorvastatin delivery after SCI significantly modulates pathway activation

Our next aim was to uncover the pathways affected by delayed delivery of atorvastatin after SCI. To elucidate these pathways, we employed Ingenuity Pathway Analysis software (IPA, Qiagen), which incorporates both the magnitude and direction of gene expression changes, along with their statistical significance, to determine pathway activation or deactivation. Our methodology involved a comprehensive examination of the complete list of 4909 DEGs associated with SCI meeting the indicated thresholds of log_2_(fold change) and FDR values (Fig. 4A). Next, IPA pathways meeting the criteria of a |Z-score| > 1.96, p ​< ​0.05 for at least one SCI vs. Uninjured comparison were enumerated (Supplementary Table 2). This unbiased approach allowed us to identify the specific pathways that underwent modulation in response to the two experimental SCI conditions relative to the Uninjured Control group and visualize differences in their activation.

To examine how atorvastatin treatment broadly alters gene expression pathways following SCI, we categorized the complete list of IPA terms in Supplementary Table 2 into two groups based on the Z-score differences between SCI-shared genes and atorvastatin-unique genes. The first group included the top 10 pathways where atorvastatin-unique DEGs showed increased activation of pathways initially triggered by SCI-shared genes (Fig. 4D). Pathway changes attributable to atorvastatin-unique genes included relative increases in liver-associated Apelin Signaling, mitochondria-associated Oxidative Phosphorylation and TCA Cycle, and the Endocannabinoid Developing Neuron Pathway. Among other increases, we also observed that atorvastatin treatment increased activation of the Superpathway of Cholesterol Biosynthesis. The second group of pathways included the top 10 for which atorvastatin-unique DEGs significantly suppressed activation of pathways driven by SCI-shared genes (Fig. 4E). Among other decreases, we observed that atorvastatin-unique genes drove relative decreases in Mitochondrial Dysfunction, as well as in MicroRNA Biogenesis and Actin Cytoskeleton signaling. Together, these initial pathway analyses reveal how delivery of atorvastatin initiated 2 ​wk after SCI and continued for an additional 4 ​wk impacts gene expression and highlight specific biological pathways that could be pivotal for the improvements in sensorimotor recovery observed.

**Fig. 5:**
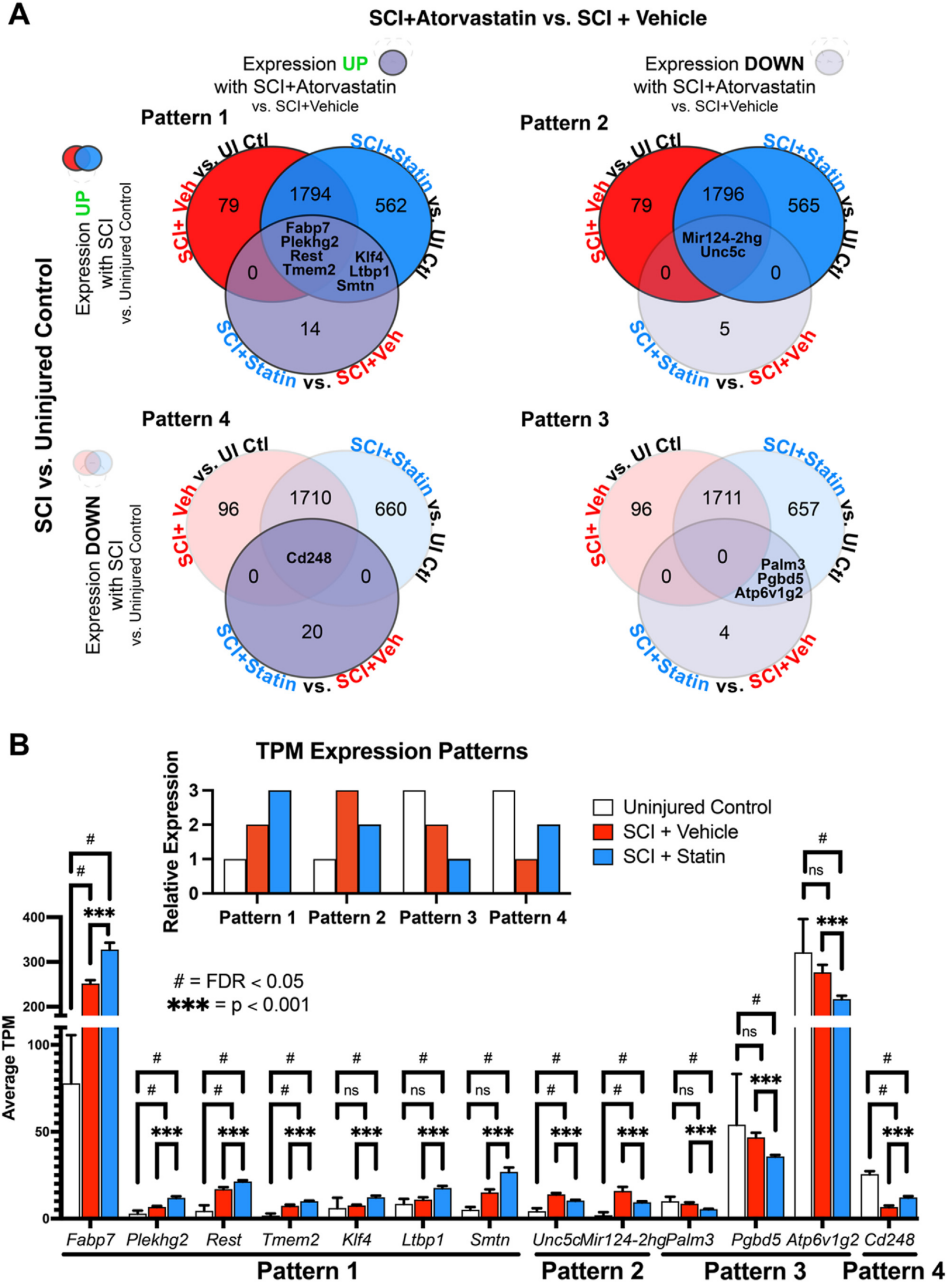
**Delayed atorvastatin delivery after SCI modulates a subset of genes beyond vehicle treatment.** (A) Venn diagram analysis of gene expression patterns after SCI, demonstrating unique and shared transcriptional changes due to atorvastatin and vehicle treatment. Pattern 1: Genes activated after SCI and further activated by atorvastatin treatment. Pattern 2: Genes activated after SCI with activation abrogated by atorvastatin treatment. Pattern 3: Genes downregulated after SCI and further downregulated by atorvastatin treatment. Pattern 4: Genes downregulated after SCI with downregulation abrogated by atorvastatin treatment. (B) Expression profiles of the 13 DEGs of interest from overlapping Venn Diagram analyses in (A), presented in transcripts per million (TPMs), illustrating the modulatory effects of atorvastatin on genes altered by SCI. Inset shows the four relative TPM expression patterns observed for the 13 genes identified in (A). Abbreviations in (A) and/or (B): “SCI ​+ ​Veh” ​= ​SCI ​+ ​Vehicle; “SCI ​+ ​Statin” ​= ​SCI ​+ ​Atorvastatin; “UI Ctl” ​= ​Uninjured Controls. Significance thresholds for comparisons: FDR <0.05 for SCI ​+ ​Atorvastatin vs. Uninjured Control and SCI-Vehicle vs. Uninjured Control; “ns” ​= ​not significant at FDR <0.05; p ​< ​0.001 for SCI ​+ ​Atorvastatin vs. SCI-Vehicle. Values represent mean ​± ​SEM. ^#^FDR <0.05; ∗∗∗p ​< ​0.001.

To identify the genes driving the impact of atorvastatin within the gene set influenced by SCI, we extended the Venn Diagram analysis to include the direct comparison of SCI ​+ ​Atorvastatin to SCI ​+ ​Vehicle (Fig. 5A). This was done to specifically isolate the effects of delayed delivery of atorvastatin on gene expression within the context of SCI and compared to the vehicle-treatment. Our approach entailed overlapping the DEG lists from different comparisons, focusing on genes either upregulated or downregulated due to SCI and further modulated by atorvastatin or vehicle treatment (Fig. 5B). This analysis yielded four distinct scenarios:

• Pattern 1: Genes upregulated in one or both SCI conditions and further upregulated by atorvastatin (SCI ​+ ​Vehicle ↑/SCI ​+ ​Atorvastatin ↑↑)
• Pattern 2: Genes upregulated in both SCI conditions but with lessened upregulation with atorvastatin (SCI ​+ ​Vehicle ↑↑/SCI ​+ ​Atorvastatin ↑)
• Pattern 3: Genes downregulated by both SCI conditions and further downregulated by atorvastatin (SCI ​+ ​Vehicle ↓/SCI ​+ ​Atorvastatin ↓↓)
• Pattern 4: Genes downregulated uniquely by SCI ​+ ​Atorvastatin vs. Uninjured Control but with significantly greater expression than SCI ​+ ​Vehicle treatment (SCI ​+ ​Vehicle ↓↓/SCI ​+ ​Atorvastatin ↓).

A key observation from the Venn Diagram overlay of gene lists in each of these patterns was the absence of DEGs exclusively associated with the SCI ​+ ​Vehicle group compared to the Uninjured Control (Fig. 5A, all patterns: overlap between SCI ​+ ​Veh vs. UI Ctl and SCI ​+ ​Statin vs. SCI ​+ ​Veh, no genes listed), which contrasts with the SCI ​+ ​Atorvastatin vs. SCI ​+ ​Vehicle comparisons (Fig. 5A, Patterns 1 and 3: overlap between SCI ​+ ​Statin vs. UI Ctl and SCI ​+ ​Statin vs. SCI ​+ ​Veh, 3 genes listed per pattern). This finding aligns with expectations, indicating no significant impact of the vehicle treatment on SCI and alternatively an impact of the delayed delivery of atorvastatin, supporting sensorimotor outcomes. Additionally, we did not identify any instances where post-SCI treatment with vehicle or atorvastatin resulted in DEG changes in opposite directions, although some genes did exhibit such patterns with less stringent thresholds—not considered in this analysis. Our analysis did identify 13 DEGs initially altered by SCI and with significantly different expression between atorvastatin and vehicle-only treatments (Fig. 5A, Patterns 1–4). The transcript-per-million expression values of these genes are shown in Fig. 5B, providing a simplified view of their transcriptional response to SCI across treatment groups.

In our final phase of analysis, we aimed to pinpoint the pathways influenced by the unique set of 13 DEGs that exhibited modulation in response to delivery of atorvastatin treatment initiated at a chronic time point following SCI (Fig. 5B). Given the limited number of genes in this subset, IPA core analysis did not yield statistically significantly modulated pathways. We therefore turned to Gene Ontology (GO) analysis, which utilizes only the list of gene names and offers a broader catalog of pathways for exploration, without considering the magnitude or statistical significance of expression changes. Our initial GO analysis with PANTHERdb (PANTHER 16.0, https://pantherdb.org/) revealed two PANTHER GO-Slim Biological Processes: “Fatty Acid Transport” (driven by *Fabp7*) and “Axon Guidance” (driven by *Unc5c*)—predicted as significantly impacted by atorvastatin treatment beyond the effects of SCI alone (both ps ​< ​0.05, Supplementary Table 3A).

For additional context, we performed additional GO analysis on the set of 13 genes using DAVID (v2024q2), which provides a more fine-grained assessment of gene-pathway relationships (Supplementary Table 3B). DAVID analysis identified enrichments of the biological processes of Somatic Stem Cell Population Maintenance driven by *Rest* and *Klf4*, Negative Regulation of Gene Expression via genes *Mir124-2hg*, *Rest*, and *Klf4*, and the molecular function of Calcium Ion Binding via *Cemip2* (*Tmem2*), *Cd248*, and *Ltbp1* (all ps ​< ​0.05). Finally, DAVID highlighted Neuronal Cell Body as a key cellular compartment involved, significantly enriched by differential expression of *Fabp7*, *Unc5c*, and *Ltbp1* (p ​< ​0.05). These pathway analyses provided additional insights into the specific biological processes, subcellular location, and gene drivers influenced by atorvastatin in the context of chronic SCI, providing candidate therapeutic targets for future study.

## Discussion

Our study is the first, to our knowledge, to demonstrate the benefits of atorvastatin in enhancing sensorimotor recovery in mice when initiated during the chronic recovery phase following SCI, a period previously characterized by heightened inflammation [59], reduced functional plasticity [60], and plateaued recovery [61]. Unlike earlier investigations where atorvastatin was administered within one day of injury—often with limited or no observed benefits [34]—our findings reveal that starting daily atorvastatin treatment two weeks post-injury significantly improves functional outcomes. We also provide thorough transcriptomic analyses of SCI and identify a significant modulatory effect of atorvastatin on multiple pathways, including several supporting neural repair and mitochondrial function. Additionally, our study uniquely identifies a small set of candidate gene drivers of recovery, including several genes previously implicated in CNS development or repair. Together, these findings provide impetus for future studies of atorvastatin in facilitating recovery in chronic SCI in humans, particularly where already indicated for cardiovascular protection, and of the mechanisms driving the pro-regenerative phenotype observed here.

Our findings not only corroborate prior research documenting the beneficial effects of statins on spinal cord injury recovery in murine models, such as those reported by Déry et al. (2009) [26], Han et al., (2011) [24], and others [62], but importantly extend the known therapeutic window of initiating statin administration, specifically atorvastatin, to the chronic period. To our knowledge, the latest initiation of atorvastatin treatment previously reported in murine contusion or compression SCI models was within 1 ​d of injury [25,26,[29], [30], [31], [32], [33], [34]]. Thus, our study newly indicates a benefit of daily atorvastatin treatment initiated in the chronic SCI recovery period for sensorimotor function as late as 2 ​wk post-injury in mice.

Our study provides a new transcriptomic dataset of murine recovery from SCI in C57BL/6J female mice 6 ​wk post injury (GSE271662, deposited in GEO DataSets) and transcriptomic analyses of DEGs altered by post-SCI atorvastatin, highlighting candidate transcriptomic mechanisms that may support enhanced sensorimotor recovery. Specifically, pathways associated with neural regeneration, such as Fatty Acid Transport [20,63,64] and Axon Guidance [65] were notably modulated. Interestingly, we also observed indications of improved mitochondrial function—enhanced activation of Oxidative Phosphorylation and TCA Cycle pathways and reduced Mitochondrial Dysfunction signaling. Together, these pathway changes indicate that delayed delivery of atorvastatin may exert protective effects on mitochondrial integrity post-injury, possibly critical post-SCI for repair, reestablishment, and maintenance of neural pathways for rapid and effective sensorimotor transmission [[66], [67], [68]]. Within the context of atorvastatin treatment during the chronic period of SCI recovery, these initial pathway analyses highlight both known mechanisms, such as fatty acid transport, as well as less researched mechanisms, such as mitochondrial function, by which delayed treatment may benefit sensorimotor recovery.

Gene-level analyses revealed precise modulatory effects of 13 genes regulated by delayed delivery of atorvastatin post-SCI, indicating nuanced mechanistic roles for the drug in neural repair. Atorvastatin modulated genes such as *Fabp7* (further upregulating post-SCI activation) and *Unc5c* (abrogating post-SCI activation), which drive, respectively, the Fatty Acid Transport and Axon Guidance pathways identified in the PANTHER GO-Slim pathway analyses. Heightened expression of *Fabp7* [[69], [70], [71], [72], [73]] or moderate-to-high expression of *Unc5c* [[74], [75], [76], [77], [78], [79], [80]], independently, have previously been reported essential for normal CNS development, function, or repair.

Additional GO analyses with DAVID highlighted *Rest* [[81], [82], [83], [84], [85], [86], [87]] and *Klf4* [[88], [89], [90]], both upregulated with atorvastatin and with prior known neuroprotective effects, as both driving Somatic Stem Cell Population Maintenance and Negative Regulation of Gene Expression. The amplified expression of these genes further supports atorvastatin’s potential to favorably modulate cellular pathways integral to stem cell maintenance and gene expression regulation when delivered at a chronic time point after SCI. We note, importantly, that GO analysis only highlights previously established associations, and the absence of a significant GO term for other genes among the 13 analyzed does not imply lesser involvement in promoting SCI recovery. Taken together, these findings offer a more nuanced understanding of atorvastatin's role in enhancing neuroprotective responses and identify novel targets for future interventional studies to promote recovery after chronic SCI.

Despite these promising findings, implications for translation require careful consideration. While atorvastatin shows potential to promote recovery when delivery initiation is delayed to a chronic time point after SCI, factors such as optimal dosing [38], timing of administration [37,91], and potential side effects [16] require thorough investigation in future studies. Given the complexity of SCI pathophysiology and atorvastatin's broad biological impacts, it is crucial to delineate the specific mechanisms through which atorvastatin aids sensorimotor recovery. Recognizing these mechanisms is particularly significant, as statins are widely prescribed to mitigate cardiovascular disease—a leading cause of death among the general population and notably elevated among individuals with SCI [92,93]. A more nuanced understanding of atorvastatin's mechanistic functions in SCI recovery may provide additional benefits by optimizing the conditions under which statins are administered post-injury, potentially enhancing later-stage neuroprotective or neuro-regenerative effects [10]. Within the context of the injured spinal cord, this may require additional investigations of the drug’s well-documented effects on lipid metabolism [94,95] and inflammation modulation [[96], [97], [98]], or exploring candidate effects newly reported here on mitochondrial integrity, axon guidance, or stem cell maintenance.

Our study on the effects of atorvastatin post-SCI reveals novel insights with therapeutic potential but is constrained by several limitations. The absence of a statin-treated uninjured control group and the use of only female mice restrict, respectively, our ability to isolate atorvastatin’s effects from general SCI responses and limit the generalizability of our findings. Future studies should comparatively study the effect of atorvastatin in the intact spinal cord to distinguish the drug's general effects from those specific to the SCI context. Additionally, in the current study, we delivered Atorvastatin via i.p. injection since this offered certain advantages, such as consistent delivery [50] and limited potential stress and injury from repeated oral gavage [48]. Given the promising impact on neurobehavioral outcomes in our model of chronic SCI, future efforts to study the impact of oral atorvastatin and optimal dosing will be essential to future clinical translation.

Having a small Uninjured Control group (n ​= ​2) additionally limited our statistical power, possibly limiting detection of DEGs in the comparative SCI conditions. Including additional uninjured samples in future studies may enhance the detection of more subtle transcriptomic differences. Importantly, however, PCA showed that the two uninjured mice clustered tightly and were distinctly separated from injured samples (Fig. 3E). Additionally, despite the small uninjured group, we detected over 3500 differentially expressed genes in the comparison of SCI to Uninjured (Fig. 4A) with rigorous significance and fold-change thresholds (FDR <0.05 and log_2_(fold change) ​> ​0.322, respectively), but only 28 in the comparison of SCI ​+ ​Atorvastatin to SCI ​+ ​Vehicle with a more relaxed significance threshold (p ​< ​0.05), indicating the importance of the larger SCI groups and the ∼40 million read count/sample. These factors strengthen our confidence in the findings despite the small uninjured group.

The use of a mouse model, while a useful mammalian model, may independently limit or complicate translatability of results due to physiological differences from SCI in humans, potentially addressed in future research using humanized-mouse models [99,100] or studies in larger animal models [[101], [102], [103]]. Additionally, our transcriptomic data alone does not capture all cellular responses such as epigenetic changes, protein dynamics, or other microenvironmental changes, and candidate targets must be validated in tissue sections and/or in vitro through functional assays in murine or human-derived cells. To conclusively establish the efficacy of atorvastatin for treating spinal cord injuries in humans and identify its key transcriptomic mechanisms, future research will need ultimately to include well-designed clinical trials. These studies should incorporate diverse populations to determine the optimal treatment parameters and validate the preliminary findings presented here.

The current study focused on the impact of atorvastatin on sensorimotor outcomes, including gait and strength, in addition to gene expression profiles in the chronically injured spinal cord. We expect that the improvements in sensorimotor function observed, as well as the key molecular changes elicited in the spinal cord itself with delayed delivery of atorvastatin after SCI will provide key rationale for future efforts to examine a wider range of behavioral outcomes, such as neuropathic pain and cognition.

In summary, while our results are promising and suggest atorvastatin as a potential therapeutic agent for chronic SCI recovery, further research is required to understand the full scope of its benefits and limitations. Future studies should aim to explore the optimal therapeutic window, dosing strategies to maximize recovery while minimizing adverse effects, and possible adjunctive benefits with other cellular [[104], [105], [106]] and/or physical rehabilitation [105] therapies. Given atorvastatin’s prior established safety profile and efficacy in the chronic SCI model tested here, we conclude that this statin is a promising candidate for further study as part of existing SCI treatment regimens, especially where already indicated for cardiovascular health and when initiated in the chronic recovery period.

## Data availability statement

The raw and processed datasets and a detailed description of the data-processing pipeline utilized for this study are available at the GEO repository: https://www.ncbi.nlm.nih.gov/geo/query/acc.cgi?&amp;acc=GSE271662.

## Credit Author Statement

**Samuel Buchl**: Conceptualization, Methodology, Data Curation, Software, Formal Analysis, Writing - Original Draft, Review & Editing, Visualization.

**Ha Neui Kim**: Methodology, Investigation, Formal Analysis, Writing - Review & Editing.

**Benjamin Hur**: Conceptualization, Methodology, Software, Formal Analysis, Data Curation, Writing – Original Draft, Review & Editing.

**Whitney Simon**: Methodology, Investigation, Writing – Original Draft, Review & Editing.

**Monica Langley**: Formal Analysis, Writing - Review & Editing.

**Jaeyun Sung**: Conceptualization, Methodology, Formal Analysis, Supervision, Writing - Review & Editing.

**Isobel Scarisbrick**: Conceptualization, Methodology, Supervision, Project Administration, Funding Acquisition, Resources, Writing - Original Draft, Review & Editing.

## Declaration of Generative AI and AI-assisted technologies in the writing process

During the preparation of this work, the author S. C. Buchl utilized ChatGPT (GPT-4, OpenAI) during the final editing stages to improve word choice and readability. After using this tool, the authors reviewed and edited the content as needed and take full responsibility for the content of the publication.

## Funding statement

This work was supported by R01NS120877 from the National Institutes of Health, the Minnesota State Spinal Cord Injury and Traumatic Brain Injury Research Grant Program, and by the Mayo Clinic Center for Biomedical Discovery. The funders had no role in study design, data collection, or analysis.

## Declaration of competing interest

The authors declare that they have no known competing financial interests or personal relationships that could have appeared to influence the work reported in this paper.

## Appendix A Supplementary data

Supplementary Table 1 **Differentially expressed genes in SCI conditions.** A table of the most significantly up- and down-regulated genes in SCI conditions, comparing SCI ​+ ​Vehicle and SCI ​+ ​Atorvastatin groups against Uninjured Controls. Top-10 genes found in both comparisons are bolded, forming the list of genes analyzed in Fig. 3D. Genes are ranked by magnitude of log_2_(fold change) and Benjamini-Hochberg adjusted p values (False Discovery Rate, FDR), with accompanying TPM values for individual replicates. **NOTE**: Included as “**SuppTable1.xlsx.”** Supplementary Table 1

Supplementary Table 2 **Comparative activation of pathways by atorvastatin or a vehicle control following SCI.** This table illustrates the differential activation of pathways in SCI mice treated with atorvastatin or the vehicle-only compared to Uninjured Controls. Ingenuity Pathway Analysis (IPA) identifies how canonical pathway activation changes with post-SCI atorvastatin treatment versus Uninjured Controls or vehicle-only treatment versus Uninjured Controls. Z-score differences were calculated as (Atorvastatin-unique genes' pathway Z-score - SCI-Shared genes' pathway Z-score). Only pathways with at least one comparison (SCI condition vs. Uninjured Control) showing a Z-score magnitude greater than 1.96 (p ​< ​0.05) were included in this table. A pseudovalue of 0.000001 was substituted for “N/A” values to enable Z-score calculations across all pathways. The following Δ|Z-score|: p-value pairings were utilized in the corresponding panels Fig. 4D-E: ∗∗∗Δ|Z-score| > 3.29: p ​< ​0.001; ∗∗Δ|Z-score| > 2.58: p ​< ​0.01 **NOTE**: Included as “**SuppTable2.xlsx.”** Supplementary Table 2

Supplementary Table 3 **Post-SCI atorvastatin treatment modulates multiple pathways pertaining to CNS injury and repair.** (A) PANTHER GO-Slim (16.0) Biological Process analysis for the 13 differentially expressed genes from Fig. 5, identifying significant pathways relevant to CNS repair. **(B)** DAVID (v2024q2) GO analysis for the same set of genes, including significant pathways across Biological Processes, Molecular Functions, and Cellular Compartments. For both (A) and (B), pathways were denoted as significant at a threshold of p ​< ​0.05. **NOTE**: Included as “**SuppTable3.xlsx.”** Supplementary Table 3
